# A de novo *SCN8A* heterozygous mutation in a child with epileptic encephalopathy: a case report

**DOI:** 10.1186/s12887-019-1796-9

**Published:** 2019-11-01

**Authors:** Kao-Min Lin, Geng Su, Fengpeng Wang, Xiaobin Zhang, Yuanqing Wang, Jun Ren, Xin Wang, Yi Yao, Ying Zhou

**Affiliations:** 1 Department of Functional Neurosurgery, Xiamen Humanity Hospital, Xiamen, 361000 Fujian China; 2 Department of Neurosurgery, The People’s Hospital of Rizhao, Jining Medical University, Rizhao, 276826 Shandong China; 3 Neuromedicine Center, the 174th Hospital of Chinese People’s Liberation Army, Affiliated Chenggong Hospital of Xiamen University, Xiamen, 361004 Fujian China; 4 National Institute for Data Science in Health and Medicine, School of Information Science and Engineering, Xiamen University, Xiamen, 361101 Fujian China; 5 Fujian Provincial Key Laboratory of Neurodegenerative Disease and Aging Research, Institute of Neuroscience, School of Medicine, Xiamen University, Xiamen, 361102 Fujian China; 6 Division of Epilepsy Surgery, Shenzhen Children’s Hospital, No.7019 Yi-tian Road, Fu-tian District, Shenzhen, 518026 Guangdong China; 7 National Institute for Data Science in Health and Medicine, School of Medicine, Xiamen University, 4221-120 South Xiang’an Road, Xiang’an District, Xiamen, 361102 Fujian China

**Keywords:** Epileptic encephalopathy, Missense mutation, *SCN8A*, Targeted exome sequencing

## Abstract

**Background:**

Epilepsy is a complex disorder caused by various factors, including genetic aberrance. Recent studies have identified an essential role of the sodium channel Nav1.6, encoded by the gene *SCN8A*, in epileptic encephalopathy.

**Case presentation:**

Using parent-offspring trio targeted-exome sequencing, we identified a de novo heterozygous missense mutation c.3953A > G (p.N1318S) in *SCN8A* in a 3-year-and-9-month Chinese female patient with early infantile epileptic encephalopathy and a normal magnetic resonance imaging of the brain.

**Conclusions:**

This de novo mutation was only detected in the patient but not in her parents. Bioinformatic analysis indicates the pathogenicity of this mutation. Administration of the sodium channel blocker well controlled seizures in the patient. Therefore, we recommend trio targeted-exome sequencing as a routine method for pathogenic variant screening in patients with intractable epilepsy and a normal MRI.

## Background

Epilepsy is one of most common pediatric neurologic disorders. The prevalence rate is estimated 12/1000 in pediatric patients. One third of them shows pharmacoresistance, and 40% of patients who are younger than 3 years are related to the epileptic encephalopathy [1]. Epileptic encephalopathy (EE) refers to a heterogenous group of epileptic disorders, characterized by intractable seizure, impairment and regression of cognitive and behavioral functions [1, 2]. The causes of EE include structure anomalies, inborn errors of metabolism and genetic insults. The exploration of numerous genetic variants is attributed to wide-spreading applications of next-generation sequencing [3], including ion channel mutations. Voltage-gated sodium channels are responsible for the initiation and propagation of action potentials. Malfunctions of sodium channels are involved in epileptic seizures [4]. Here we reported a patient with infantile EE probably caused by a de novo missense mutation of *SCN8A* (c.3953A > G, p.N1318S).

## Case presentation

**Fig. 1:**
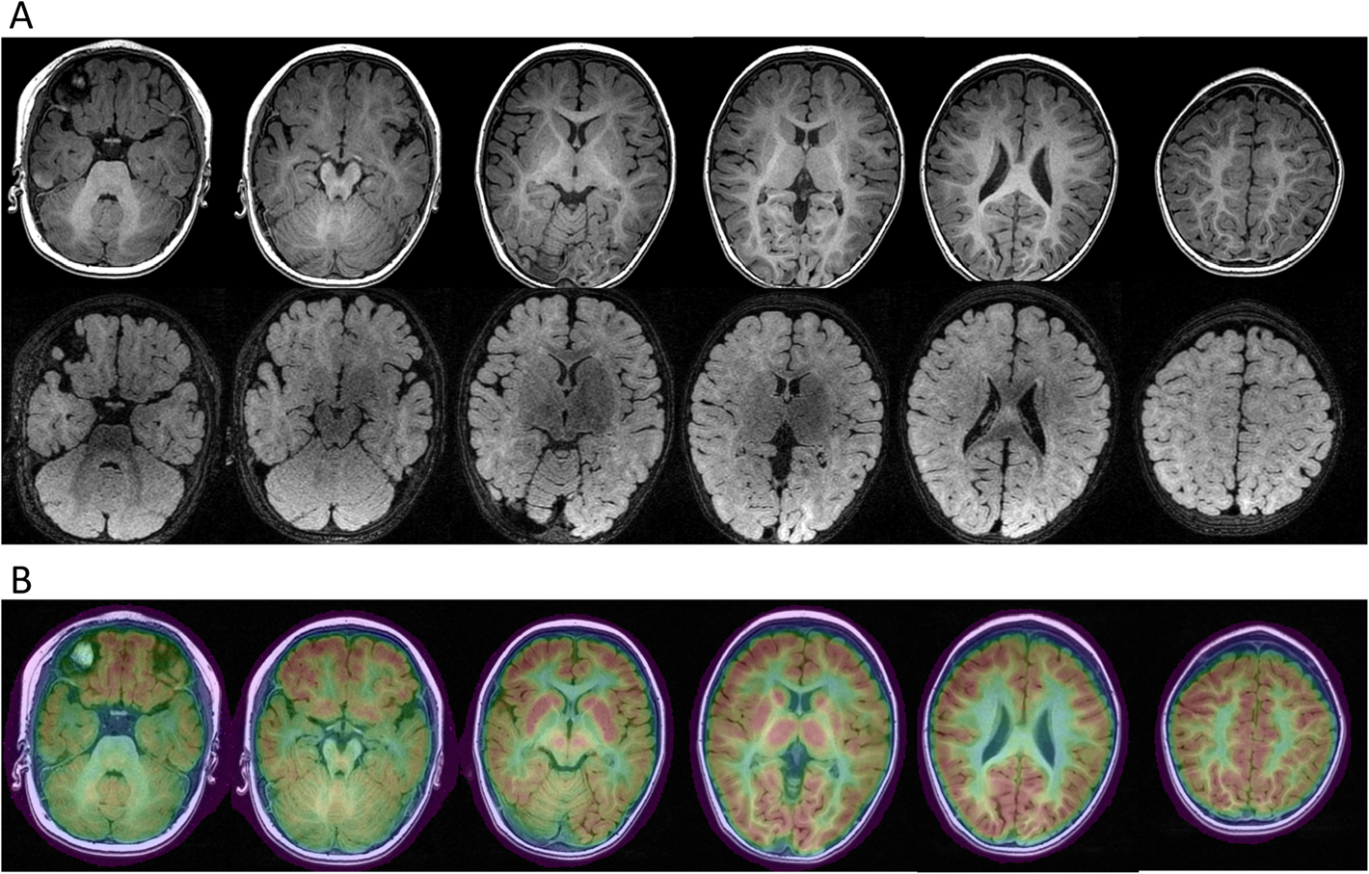
Brain MRI and FDG-PET of the patient. **a** Brain MRI in axial view with T1/T2-FLAIR series shows unremarkable findings. **b** FDG-PET fusion with MRI shows unremarkable focal hypometabolism

**Fig. 2:**
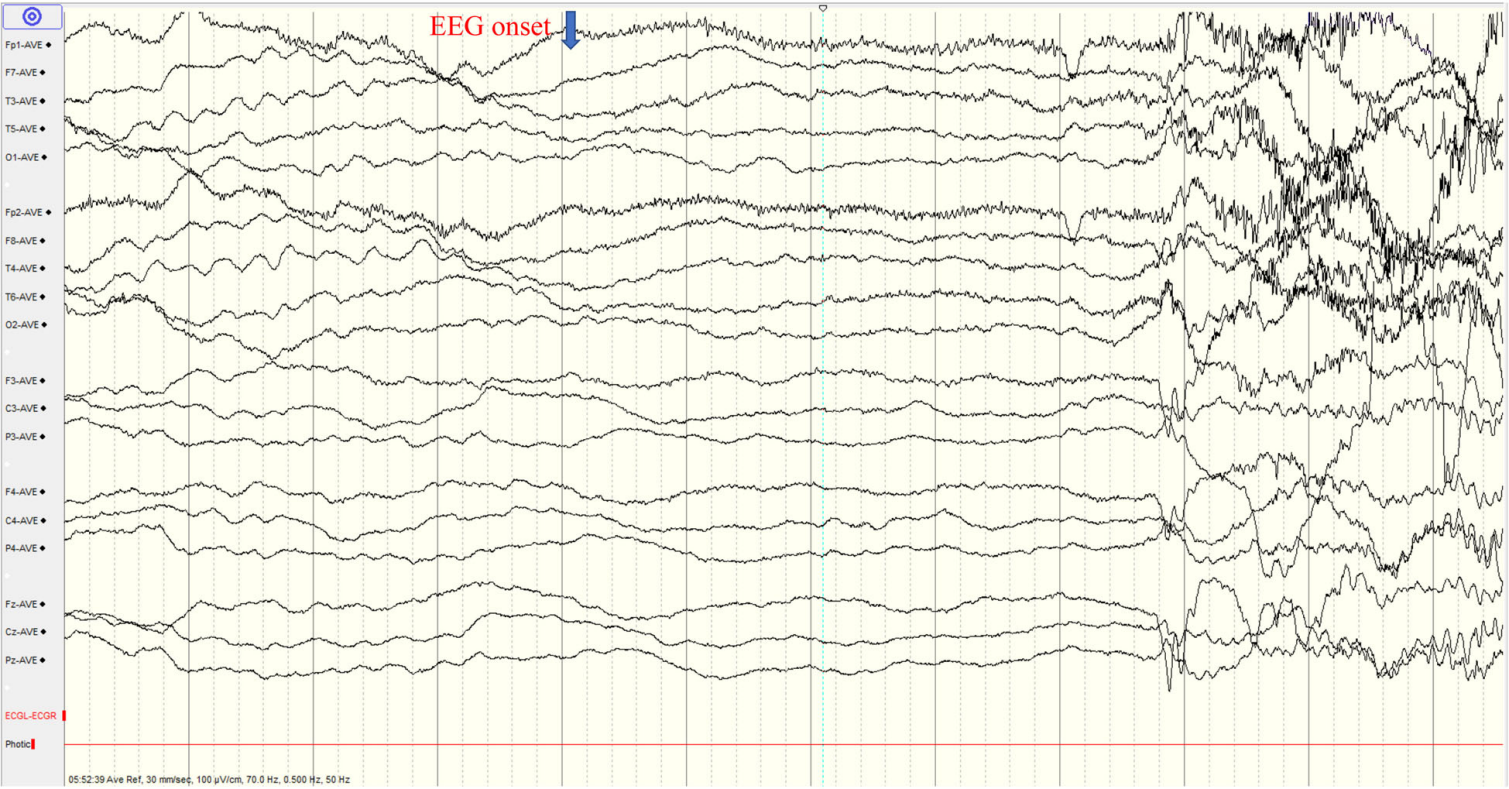
EEG at ictal onset demonstrates generalized electrical attenuation with superimposed fast activity and muscle artifacts. (paper speed: 30 mm/sec, sensitivity: 10 μV/mm, band pass: 0.5–70 Hz, notch filter: 50 Hz)

The 3-year-and-9-month old girl was born at term with uneventful maternal pregnancy, delivery and family history. Her birth weight and body length were both within normal limits. She had the first afebrile seizure at night sleep presented as generalized tonic-clonic seizure lasting for 1–2 min while she held the normal developemtal milestone of social smiling at her age of 2 months. Topiramate was prescribed, but seizures still happened, until oxcarbazepine monotherapy employed that seizure temporarily resolved for half a year. Later, afebrile seizure flared up and the frequency was around 1–2 fits per month, even several convulsive status epilepticus. She came to our attention 1 year later. Physical and neurologic examinations, including the head circumference, muscle tone and deep tendon reflex, and the laboratory data were all unremarkable. Metabolic survey of amino and organic acids, brain magnetic resonance imaging (MRI) and fluorodeoxyglucose (FDG)-positron emission tomography (PET) demonstrated unremarkable findings (Fig. 1). The test of Wechsler Preschool and Primary Scale of Intelligence (WPPSI-IV) showed mild cognitive impairment (full scale IQ = 66). Interictal electroencephalogram (EEG) revealed normal background activity without obvious epileptiform discharge. Ictal video EEG recorded the seimology of generalized tonic, tonic-clonic seizures, and generalized electrical decrement with superimposed fast activity at EEG ictal onset (Fig. 2). Phenytoin (5 mg/kg/day), valproic acid (20 mg/kg/day), levetiracetam, clonazepam were ever tried, but there was little benefit. Meanwhile, short-term seizures that were provoked by low-grade fever developed then.

Genetic counselling was recommended because her seizures were poorly controlled. High-throughput sequencing of exons of disease-causing genes were performed on the patient and her parents. Genomic DNA extraction and library preparation followed the standard Illumina protocols (Illumina, San Diego, USA) with minor adaptation provided by Joy Orient (Joy Orient Translational Medicine Research Center Co. Ltd., Beijing, China). Agilent Bioanalyzer 2100 (Agilent Technologies, USA) was used for quality control of DNA size distribution and enrichment. Target capturing was performed using Roche (Roche AG., Basel, Switzerland) product customized by Joy Orient, which uses 91,867 probes to capture total 7,465,978 bp of exons regions of 3372 genes that are potentially associated with 4213 known Mendelian genetic diseases. A HiSeq2500 sequencer was used to perform high-throughput sequencing. Exon-enriched DNA was sequenced by the Illumina hiseq2500 platform following the manufacturer’s instructions (Illumina). Raw image files were processed by the BclToFastq (Illumina) for base calling and generating the raw data. The low-quality variations were filtered out using the quality score ≥ 20 (Q20). The sequencing reads were aligned to the NCBI human reference genome version hg19 using BWA. Samtools and Pindel were used to screen SNP and indel of the sequence. All genetic variants were screened by pathogenicity, mode of inheritance and clinical phenotypes **(**Table 1**).**

**Table 1:** Workflow of filtering the pathogentic variant

	Variants No.	Note
GATK Haplotype (Total variants from a trio targeted-exome sequencing)	20,076	total variants from a parent-proband trio targeted sequencing
1st round of Filtering criteria		variants were excluded using a hierachy of levels of filtering criteria
proband wild-type	5971	exclude variants only in the parants but not in the proband
intron> 30 bp	2490	
AF < 0.2 or AD< 4 or MQ < 35	1124	
SSR > =7 & AF < 0.3 indel	1178	
indel> 50 bp	4	
After 1st round of filtering		
Variants No. in the proband	9309	a five-tier system of classification for variants (ACMG guidelines, 2015)
Benign	8775	
Likely benign	158	
Uncertain significance	346	
Likely pathogenic	23	
Pathogenic	7	
2rd round of filtering criteria		
Pathogenic/Likly Pathogenic/Uncertain significance & OMIM	368	overlap these 3 types of variants with OMIM
segregation analysis	20	a specific variant in the target gene is observed to segregate with a phenotype or disease
variants which are associated with patient’s clinical phenotype	1	SCN8A:c.3953(exon22)A > G, p.Asn1318Ser, AF = 70/174 = 0.4
AF: allele frequency		
AD: allele depth		
MQ: Mapping quality		

**Fig. 3:**
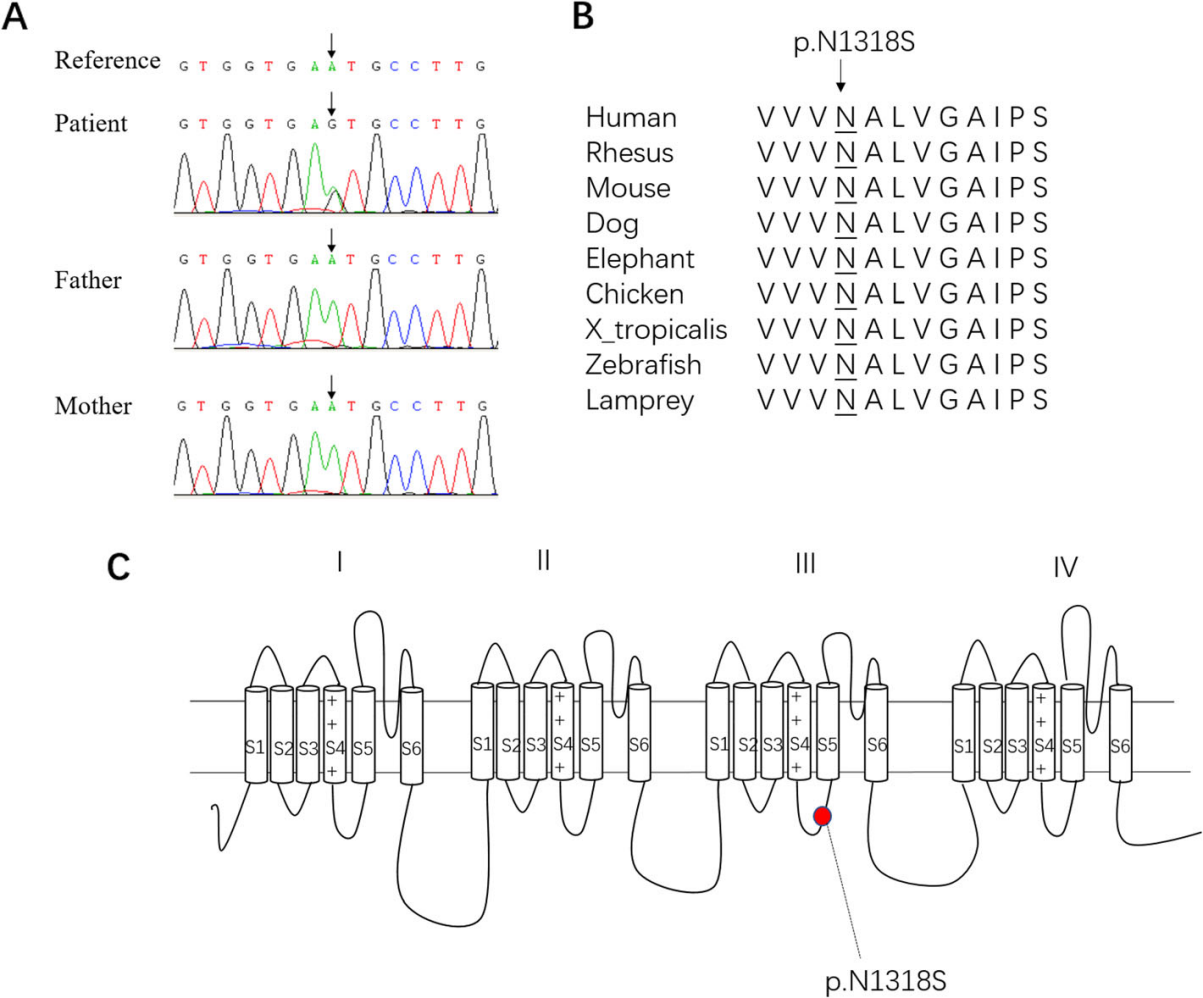
Characterization of *SCN8A* (c.3953A > G) mutation. **a** Sanger sequencing confirms *SCN8A* (c.3953A > G) mutation. **b** The down-stream altered amino acid caused by the missense mutation is in a highly-conserved area. **c** This mutation is located in the internal S4-S5 linker of the DIII of SCN8A protein

Importantly, a de novo heterozygous mutation c.3953A > G in *SCN8A* (the coverage of the variant (DP) is 174, allele frequency (AF) = 70/174 = 0.4) was identified and confirmed by Sanger Sequencing (Fig. 3a). The detailed information of this variant is as the followings: *SCN8A* (NM_014191.4), missense mutation, c.3953A > G (exon22), p.N1318S (de novo), location: chr12, 52,180,336. This missense mutation causes an amino acid substitution of an asparagine residue with a serine residue (p.N1318S) which occurs at a highly conserved LINKER position between the S4 and S5 segments in the third transmembrane domain (DIII) of SCN8A protein (Fig. 3b and c). This mutation site is absent from ClinVar [5] and HGMD Public [6]. It is neither recorded in the Exome Aggregation Consortium (ExAC) [7], which contains exome sequencing data from 60,706 unrelated individuals, nor in our 100 in-house controls. The substitution p.N1318S is predicted to be highly deleterious by bioinformatic tools, which predict possible impact of an amino acid substitution on the structure and function of a human protein, such as Polyphen2 [8] (HumDiv score = 1; HumVar score = 0.998), SIFT [9] (score = 0), Mutation Taster [10] (score = 1) and a comprehensive index CADD phred score (C score = 23.2) [11] (Table 2).

**Table 2:** Evaluation of possible impact of c.3953A > G, p.N1318S mutation of *SCN8A* by different bioinformatic prediction tools

Tools	PolyPhen2		CADD		SIFT		Mutation Taster	
	Prediction	Score	Prediction	Score (cutoff = 12.37)	Prediction	Score (cutoff = 0.05)	Prediction	Probability
p.N1318S	Damaging	1.000	Deleterious	23.2	Deleterious	0.000	Disease causing	1

Since the probable causative gene mutation was found, levetiracetam was stopped, but her family reported more seizures. Accordingly, the therapeutic regimen of a sodium channel blocker, lamotrigine (5 mg/kg/day), valproic acid (24 mg/kg/day) and levetiracetam (10 mg/kg/day) were employed, then she had a second temporary seizure-resolved period of 5 months, even under high fever.

### Discussion and conclusions

SCN8A is widely expressed in the central and peripheral nerve systems during the neuronal maturation [4, 12]. It is mapped to chromosome 12q13, encoding neuronal voltage-gated sodium channel α 8-subunit Nav1.6, which forms a complex combined with β subunits to modulate current conductance [4, 13, 14]. Nav1.6 consists of four transmembrane domains (DI-DIV), each containing six segments (S1-S6). Four S4 transmembrane segments are responsible for the voltage sense which contain positively charged arginine and histidine residues. There are fast and slow inactivation phases. Fast-inactivation phase is provided by internal DIII-DIV linkers to occlude the ion-conducting pores. Slow-inactivation phase is involved in a collapse of the pore, which is composed of S5-S6 segments of four domains [4].

Missense mutations of *SCN8A* accounting for 1% of EE are associated with a wide-spectrum phenotype of heterogenous epilepsy, and *SCN8A* missense mutations are recently recognized to be associated with early infantile epileptic encephalopathy type 13, ref. [15–18] which displays multiple seizure types, including focal seizures, generalized seizures (tonic, myoclonic, absence) and epileptic spasms. These patients may experience stormy epilepsies [15, 17]. Fever rarely triggers seizures [4, 13, 17]. Severity of psychomotor delay ranges widely after seizure onset [15, 17]. MRI studies are typically normal [15]. There is no clear correlation between phenotypic severity and genetic mutations so far [1, 4]. *SCN8A* mutations lead to premature channel opening, impaired inactivation and increased persistent current. Stormy seizures are reported resulting from gain-of-function effects [4]. It could explain that some patients respond well to sodium channel blockers in halting seizures [15, 17, 19, 20].

As to our patient, she was categorized as the intermediate phenotype of EE, who manifested mild psychomotor retardation and infrequent seizure episodes, though developing stormy onset of generalized tonic and tonic-clonic seizures concomitant with normal EEG background activity [15, 17, 18]. Fever barely triggered seizures in our patient as previously reported [4, 13, 17]. MRI demonstrated no significant findings. She responds well to the sodium channel blockers. Targeted exome sequencing revealed a heterozygous missense mutation (c.3953A > G, p.N1318S) in *SCN8A* which is located in the internal S4-S5 linker of the DIII. This mutation could alter the function of the down-stream protein. Since *SCN8A* (c.3953A > G, p.N1318S) is a de novo missense mutation, it is necessary to further validate its function in the future.

## Data Availability

All available data are presented. The mutation information has been submitted to ClinVar, the ClinVar accession SCV000987318.

## Abbreviations

EE: Epileptic encephalopathy
EEG: Electroencephalogram
FDG-PET: Fluorodeoxyglucose-positron emission tomography
MRI: Magnetic resonance imaging
WPPSI: Wechsler Preschool and Primary Scale of Intelligence
